# Sexual Well-Being and Aging Patterns: Findings of a Cluster Analysis among Older Adults in Portugal and Spain

**DOI:** 10.3390/ejihpe14070134

**Published:** 2024-07-11

**Authors:** Sofia von Humboldt, Emilia Cabras, Gail Low, Isabel Leal

**Affiliations:** 1 William James Center for Research, ISPA–Instituto Universitário, Rua Jardim do Tabaco, 34, 1149-041 Lisbon, Portugal; ileal@ispa.pt; 2 Department of Education, Universidad Alfonso X EI Sabio, 28691 Madrid, Spain; ecabras@hotmail.it; 3 Faculty of Nursing, University of Alberta, Edmonton, AB T6G 1C9, Canada; gaill@ualberta.ca

**Keywords:** adjustment to aging, cluster analysis, older adults, satisfaction with life, sexual satisfaction

## Abstract

Objectives: From a cross-cultural perspective, aging well may encompass pertinent challenges in terms of adjustment, sexual well-being, and satisfaction with life in the late years. Considering the paucity of empirical data concerning cultural diversity of experiencing aging, this study aims to help fill this gap by assessing the specific patterns of sexual satisfaction, adjustment to aging (AtA), and life satisfaction with life (SwL) of older adults in Portugal and Spain. Methods: This cross-national study included 326 older adults, age 65 and older, from Portugal and Spain. Five instruments were applied: (a) Adjustment to Aging Scale (ATAS); (b) Satisfaction with Life Scale (SwLS); (c) New Sexual Satisfaction Scale-Short (NSSS-S); (d) Mini-Mental State Exam; and (e) Sociodemographic, Health and Lifestyle questionnaire. K-means cluster analysis was employed to identify and characterize the clusters considering adjustments to aging, sexual satisfaction, and life satisfaction. One-way ANOVAs were conducted to analyze differences in sexual well-being among clusters. Results: Findings indicated three clusters, which explained 77.7% (*R-sq* = 0.777) of the total variance: Cluster 1: “Most skilled” (*n* = 26, 8.0%), Cluster 2: “Least adjusted” (*n* = 115, 35.3%), and Cluster 3: “Aging strivers” (*n* = 185, 56.7%). Participants in Cluster 1 were mostly Portuguese, with high levels of AtA, sexual satisfaction, and SWL. Conversely, Cluster 2 included mostly Portuguese participants with moderate sexual satisfaction and lower levels of AtA and SwL. Participants from Cluster 3 were mostly Spanish, with moderate levels of AtA and reduced sexual satisfaction and SwL. Conclusions: This study innovates by exploring the elaborate interplay among sexual satisfaction, AtA, and SwL in a cross-cultural perspective, with implications for tailoring interventions, service planning, development, and evaluation of culturally diverse older populations.

## 1. Introduction

Countries worldwide are facing significant challenges in adapting their health and social systems to accommodate the aging of the population [1]. Portugal ranks as the third-oldest country among the 27 European Union (EU), while Spain holds the eighth position [2]. In 2021, Portugal (22.4%) and Spain (19.8%) stood out as countries with the highest proportions of people aged 65 or older in their populations [3].

The aging of the population has implied the need for a comprehensive understanding of aging, considering the complex interconnection of variables, such as adjustment to aging (AtA), sexual well-being, and overall satisfaction with life (SwL). Moreover, an inclusive approach underlines the importance of understanding the multifaceted dynamics of aging [4,5,6,7].

Old age is frequently associated with dependence, apathy, illness, and diminished sexual allure [8]. An increasing body of literature often promotes a youthful image, rather than one of aging, to emphasize sexual attractiveness. Additionally, the media frequently show ideals for beauty and sexual attractiveness by portraying young and wrinkle-free bodies [8,9]. A youthful appearance is closely tied to increased sexual desire interest and attractiveness, with diverse effects on sexual satisfaction [8,9]. Indeed, older adults frequently face ageist stereotyping of both their sexuality and their aging bodies [7,8]. Moreover, in some cultures, sexual activity is seen as energy-intensive and unnecessary for maintaining health and well-being. Indeed, some cultural standards tend to view older individuals as devoid of sexuality, emotions, and sensations [9]. Conversely, recent decades have witnessed shifting attitudes regarding sexual engagement in older life. The majority of older individuals remain sexually active, and sexual interactions and emotional intimacy significantly seem to contribute to their overall well-being [10].

Sexual well-being has been indicated as the emotional, physical, and cognitive assessment of an individual’s sexual life, including relationship quality, sexual satisfaction, activity, attraction, pleasure, intimate relationships, self-esteem, and other psychosexual factors [10,11], and it is an important component of overall well-being [9,12]. The term *sexual satisfaction* refers to the appraisal of one’s sexual life, both physically and non-physically [13].

A significant proportion of older individuals view sexual satisfaction as pivotal to a satisfying relationship and overall SwL [13]. Recent research has expanded the knowledge, revealing a connection between positive well-being and sexual satisfaction in older adulthood [14]. Moreover, literature has indicated associations between regular sexual activity and reduced relationship tension [15], as well as positive correlations between the frequency and significance of sexual experiences and sexual satisfaction for both partnered and single older adults [16,17]. Additionally, emotional intimacy, personal communication, and relationship happiness are all positively correlated with higher levels of sexual satisfaction [11].

Research about sexual satisfaction in the Spanish [18] and Portuguese [19] older populations is scarce. Almost 50% of the Spanish older adults reported being sexually activity, but sexual activity decreased with age [20]. This highlights that a significant portion of the older population still values and engages in sexual activity, emphasizing its importance despite age-related declines. Moreover, older Portuguese individuals display an interest in sexuality, and in one study, one-third of the participants expressed a need to express their sexuality [19]. Additionally, 16.6% of older participants believe they should engage in more sexual experiences, reflecting favorable attitudes toward sexuality in old age [19], indicating that many still see it as an important part of life.

In a different study, Portuguese older adults primarily associated their sexual well-being with self-reported good health and demonstrations of love. However, the unavailability of a partner emerged as the most prevalent theme (17.9%) contributing to their sexual dissatisfaction [21], suggesting that while health and emotional connection are crucial, social factors like partner availability significantly impact sexual well-being.

Among older Spanish adults, the primary reasons for low sexual well-being were the death of a spouse (31%), loss of sexual desire (29%), and health considerations (17%) [20]. For Spanish people, health status is essential apart from age, so older people who reported good health and vitality were more likely to be sexually active [20] and showed better sexual satisfaction [22]. This finding reinforces the idea that maintaining good health is vital for continued sexual activity and satisfaction in older age.

Despite recognizing the importance of sexual satisfaction and physical intimacy for older adults’ well-being, the role of sexuality in aging well has been largely overlooked [23].

Moreover, cultural norms and gender expectations also shape the sexual experiences of older individuals. For example, in Nordic cultures, sexual interactions between men and women are negotiated with relatively more equality compared to Southern cultures. Despite this, women historically occupied subordinate roles to men in these societies. The contemporary emphasis on gender equality may have empowered women to feel more in control of their sexuality, potentially leading to a shift in traditional gender dynamics where men may feel disempowered and insecure about their roles [24]. The extent to which older individuals feel comfortable expressing their sexuality and advocating for resources promoting sexual health depends on the prevailing culture and its associated gender roles, health accessibility, and affordability [24].

As people get older, SwL becomes increasingly more important [25]. Satisfaction with life may be characterized as people’s opinions about the worth or goodness of their lives, their overall level of happiness, or occasionally how satisfied they are with their lives in general. Other terms for this description include subjective well-being and happiness with life [26]. Literature also highlighted the link between sexual satisfaction and SwL in both older men and women. Across age groups, SwL is closely intertwined with contentment in one’s sex life and forms an integral aspect of SwL for older adults. Additionally, enhancing sexual satisfaction might indeed have a positive influence on the process of aging well [27].

Aging is an enduring process that requires continuous adjustment [7]. von Humboldt et al. considered AtA to be a critical aspect of the well-being of older adults [28]. Furthermore, consistent evidence indicates that older adults develop active and purposeful strategies for AtA, by including psychological factors, interactions with significant others, sexual intimacy, and social comparisons [7,29,30,31]. AtA is a dynamic process that involves the alignment of personality, social environment, and well-being, with implications for aging well [28]. Moreover, AtA, well-being and health of older individuals are affected by love, intimacy, and sexual satisfaction [7,27,32]. Considering health as the pursuit of maximum individual functionality, AtA is relevant for a health-focused approach to aging well [30].

Aging is a multifaceted process influenced by cultural, ethnic, and gender differences. Variations in the perception of well-being, satisfaction, adjustment, and sexuality between different cultures and genders are substantial [33,34]. Modern societal views of old age now acknowledge the possibility of older individuals experiencing joy, satisfaction, adjustment, vitality, and health while engaging in sexual activity; however, age-related differences in love and sexuality may be influenced by culture [33]. Despite the connection between AtA, SwL, and sexual satisfaction, there is a noticeable lack of comprehensive cross-cultural studies about the intricate relationships among these constructs [34,35].

Additionally, while there have been studies on sexual activity, there has been a lack of cross-cultural comparisons of older individuals in different countries utilizing consistent measurements, sociodemographic variables, and data collection techniques.

This study aims to address these gaps by using a cluster analysis approach to examine specific patterns of sexual satisfaction, AtA, and SwL among older adults in Portugal and Spain.

## 2. Materials and Methods

### 2.1. Participants

Our sample included 326 older people (*M* = 70.59; *SD* = 5.24). Among them, 54.3% were Portuguese and 45.7% were Spanish. The age of participants varied between 65 and 92 years old; 51.5% were female. Our sample is well educated, with 50% having a bachelor’s/master’s/doctoral degree, 12.3% having completed secondary school, and 37.7% of the individuals having only completed elementary school. Furthermore, a substantial 78.8% of participants reported good health. Less than 10% of participants disclosed recent psychological concerns, with a predominant focus on issues related to anxiety and depression. Including these demographic characteristics is essential for identifying distinct clusters and patterns in sexual satisfaction, AtA, and SwL among older adults and for providing a comprehensive understanding of the factors influencing the aging process. Table 1 lists the sociodemographic details of the participants.

Participants were recruited from senior universities, message boards, emails, community center lists, and network contacts. Different recruitment sources contributed to the sample diversification, with senior universities targeting lifelong learners, message boards and emails reaching a broader audience, and network contacts accessing existing social networks, ensuring a varied pool of participants. The eligibility criteria encompassed the following requirements: (a) Participants were required to be a minimum of 65 years old, (b) possess a clear understanding of their voluntary participation in the study, and (c) have no prior history of cognitive impairments, psychiatric or neurological disorders, substance abuse, or similar conditions. Additionally, participants were expected to possess proficiency in utilizing contemporary technologies, such as smartphones, tablets, computers, and apps. For queries or assistance, participants were encouraged to seek support either through phone or online channels. Participants were screened to ensure their eligibility based on their capacity for decision-making and cognitive abilities.

### 2.2. Instruments

#### 2.2.1. Adjustment to Aging Scale (AtAS)

The Adjustment to Aging Scale (AtAS) comprised five different subscales, and the internal reliability of the scale and its five subscales proved to be strong: sense of purpose and ambitions (0.874); zest and spirituality (0.927); body and health (0.904); aging in place and stability (0.862); and social support (0.932). Cronbach’s alpha coefficient indicated an internal consistency of 0.891 for the overall scale. Respondents used a 7-point Likert-type scale, ranging from 1, which indicates “Not at all important”, to 7, which indicates “Very important”. This measure proved good psychometric properties, and the low cross-national invariance of this measure highlights its cross-cultural character [28], further underscoring its suitability for cross-cultural assessments, aligning with our study’s aim to explore AtA across different cultural contexts.

#### 2.2.2. New Sexual Satisfaction Scale-Short (NSSS-S)

The 12-item New Sexual Satisfaction Scale-Short (NSSS-S) has a two-dimensional factor structure, assessing self-centrality and partner and sexual activity centrality. It may be utilized with individuals from both genders and has recently been verified in Spanish and Portuguese samples [36,37]. The NSSS-S is composed of five 5-point ordinal items, with higher scores on the scale indicating higher levels of sexual satisfaction. The NSSS-S shows strong psychometric qualities in terms of validity and reliability. Furthermore, internal consistency was evidenced by Cronbach’s alpha of 0.94, which assured confidence in its ability to provide robust measurements of sexual satisfaction among older adults, enhancing the reliability of our study’s findings.

#### 2.2.3. Satisfaction with Life Scale (SwLS)

The Satisfaction with Life Scale (SwLS), developed by Larsen et al. [38], was designed as a quick assessment of a person’s overall level of satisfaction with life. The SwLS has only five items but has been shown to be a useful instrument for assessing the mental population and to have high psychometric qualities [39]. The SwLS has been used in several cross-cultural studies investigating life satisfaction and SWB in general. In addition to its demonstrated validity and reliability of the life satisfaction component, the SwLS scale’s strong internal consistency (*r* = 0.78) [39,40] further supports its suitability for assessing overall life satisfaction among older adults, reinforcing the robustness of our measurement approach.

#### 2.2.4. Sociodemographic, Health and Lifestyle Questionnaire

Key sociodemographic, health, and lifestyle details of the participants were analyzed. It included information on age, gender, education level, income, employment, and marital status, among others. The health and lifestyle component of the questionnaire included the participant’s current state of health, medication lifestyle decisions, and access to medical treatment.

### 2.3. Procedure

The objectives of the study were explained to the participants. Every participant was made aware that their participation was entirely free and that they might quit at any time. Confidentiality and anonymity of the data were guaranteed. Data collection took place from 1 October to 30 December 2022. No compensation was given to respondents for completing the questionnaires. The ISPA-Instituto Universitário and the Research Ethics Committee of the William James Center for Research gave their approval to all procedures. The ethical principles outlined in the Helsinki Declaration and the Code of Ethics of the Portuguese Psychologists were followed in this study. In addition, other precautions to handle sensitive issues included ensuring anonymity and confidentiality, providing informed consent, and offering support resources for participants who may experience discomfort or distress during the study. We also employed measures to ensure the participants’ ease and privacy, such as offering the option to withdraw from the study at any time, without consequences, and providing clear communication about the nature and purpose of the research.

### 2.4. Data Analysis

The 326 respondents were divided into different groups using cluster analysis based on their scores in various measures, which included AtA (sense of purpose and ambitions, zest and spirituality, body and health, aging in place and stability, and social support), sexual satisfaction (ego-centered and partner/sexual activity centered), and SwL. The sample size was determined based on a power analysis to ensure sufficient statistical power to detect expected effect sizes in our analyses. With an error probability of 0.05, the power analysis indicated that a total sample size of 326 showed a power of 0.95, for an effect size of 0.50. The final sample size is lower than the initially proposed sample size of 400 participants, which corresponded to an approximately 20% expected attrition rate due to participants that did not match eligibility criteria or decided to withdraw from the study.

We aimed to identify distinct groups based on specific measures, and for this purpose, we used hierarchical cluster analysis with the Ward technique, which, in turn, used squared Euclidean distance as a measure of subject difference. This approach was favored over other possible methods due to its ability to handle large datasets and its capability to produce well-separated and interpretable clusters; hence, it is suitable for providing a comprehensive understanding of the underlying patterns within the data.

The *R*^2^ evaluation, as indicated by Marôco [41], was utilized to establish the correct number of clusters. The analysis identified three homogenous clusters, accounting for around 77.7% of the total variance. The classification of each subject within these clusters was then further refined using the non-hierarchical *k-Means* approach.

Following the methodology outlined in Marôco [41], the significance of each variable in each of the three clusters was assessed using the *F* statistics of the clusters ANOVA. Chi-square analysis was used to investigate how people in each of the three clusters differed in terms of sociodemographic, health, and lifestyle variables.

Additionally, mean differences between cluster groups were evaluated using Analysis of Variance (ANOVA) and Tukey HSD tests, with sexual well-being indicators as the dependent variables. Statistical significance was determined to be *p* < 0.05 for all analyses. To describe the sample, descriptive statistics were utilized. All analyses were made with IBM SPSS Statistics for Windows, version 29.

## 3. Results

This study aimed to examine specific patterns of sexual satisfaction, AtA, and SwL among older adults in Portugal and Spain.

**Figure 1:**
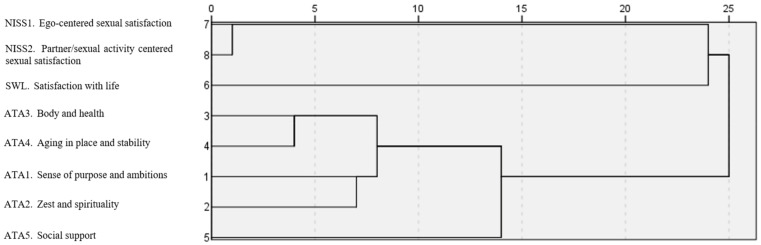
Dendrogram: Hierarchical clustering with single linkage.

Using *R*^2^ criterion, findings indicated the retention of three clusters, which together accounted for 77.7% (*R-sq* = 0.777) of the total variance. This *R*^2^ criterion indicates that approximately 77.7% of the variance of the dependent variable is explained by the independent variables in the model; that is, this model provides a robust explanation for the patterns observed in the data. The dendrogram representing the hierarchical relationships shows the three clusters (see Figure 1).

The centers of the clusters and the *F* statistics for each variable are presented in Table 2. The *F*-statistic determines whether the model as a whole is statistically significant, indicating that the observed relationships are unlikely to be random. Among the dimensions analyzed, those that most differentiate the clusters are related to AtA. Specifically, “body and health” (*F* = 309.887) has the highest differentiating effect, followed by “aging in place and stability” (*F* = 262.061), “social support” (*F* = 196.478), “zest and spirituality” (*F* = 192.719) and “sense of purpose and ambitions” (*F* = 161.274). Both ego-centered sexual satisfaction (*F* = 149.102) and partner/sexual activity-centered sexual satisfaction (*F* = 148.255) also contribute to differentiating the clusters. On the other hand, satisfaction with life (*F* = 81.302) is the dimension that least differentiates the clusters.

This study aimed to identify clusters that were characterized by varying levels of AtA, sexual satisfaction, and SwL, which supported our hypothesis regarding the intricate relationships among these constructs in the context of aging.

The three distinct clusters were retained based on participants’ scores in various dimensions. The first cluster (Cluster 1: “most skilled”; high AtA/high sexual satisfaction/high SwL) included 26 participants who exhibited relatively high levels of AtA, sexual satisfaction, and SwL. The second cluster (Cluster 2: “least adjusted”; low AtA/moderate sexual satisfaction/low SwL) comprised 115 participants with low levels of AtA and SwL and moderate sexual satisfaction. Finally, the third cluster (Cluster 3: “aging strivers”; moderate AtA/low sexual satisfaction/low SwL) included 185 participants with moderate levels of AtA and low levels of sexual satisfaction and SwL.

The results indicate that the three clusters showed specific sociodemographic features, which support our hypothesis regarding the variations in sociodemographic, health, and lifestyle factors among the participants in each of the three clusters.

In this context, chi-square tests (*χ*^2^) were performed to examine the variations in sociodemographic, health, and lifestyle traits among the participants in each of the three clusters (see Table 3).

The results of the study revealed significant differences among the three clusters in terms of nationality [*χ*^2^ (2) = 17.572, *p* < 0.001]. Cluster 1 (“most skilled”) included mostly Portuguese participants, while Cluster 3 (“aging strivers”) included mostly Spanish people. In terms of sex [*χ*^2^ (2) = 3.273, *p* = 0.195], Cluster 1 exhibits a greater prevalence of women, whereas Cluster 3 shows a higher incidence of men.

Furthermore, there were significant differences in terms of education [*χ*^2^ (12) = 45.111, *p* < 0.001]. Interestingly, participants with lower educational levels were mostly in Cluster 1 (“most skilled”). In contrast, participants with higher levels of education were mainly in Cluster 2 (“least adjusted”) and Cluster 3 (“aging strivers”). This result may indicate that factors beyond education contribute significantly to the highest levels of AtA, SwL, and sexual satisfaction, prompting further investigation to understand the additional influences that may be affecting these aspects of well-being. Significant differences were also found in marital status [*χ*^2^ (6) = 45.225, *p* < 0.001]. Cluster 1 (“most skilled”) showed more married participants than the other clusters.

In addition, being a spiritual person [*χ*^2^ (2) = 2.646, *p* = 0.266] appeared to be associated with a high level of AtA, sexual satisfaction, and satisfaction with life in Cluster 1 (“most skilled”). Participants in Cluster 2 (“least adjusted”) were more likely to report perceived poor general health [*χ*^2^ (2) = 29.556, *p* < 0.001].

Additionally, having a positive love experience in life [*χ*^2^ (2) = 43.461, *p* < 0.001] and engaging in leisure activities [*χ*^2^ (2) = 4.332, *p* = 0.115] were mostly shown by participants in Cluster 1 (“most skilled”).

Last, results indicated that the three clusters showed specific sexual well-being features, which support our hypothesis regarding the variations in sexuality-related items among the participants in each of the three clusters.

The findings of group differences are summarized in Table 4. Cluster membership was associated with significant variations in participants’ perceived overall sexual well-being [*F*_(2174)_ = 16.987, *p* < 0.001], sexual attractiveness [*F*_(2,322)_ = 23.694, *p* < 0.001], sexual openness and communication [*F*_(2,323)_ = 93.160, *p* < 0.001], and sexual satisfaction [*F*_(2,323)_ = 93.233, *p* < 0.001]. The distinct clusters identified in our study, distinguished by sociodemographic, health, and lifestyle traits and sexual well-being variables among participants, contribute to our research aims concerning the multifaceted influences on aging experiences.

Post hoc multiple comparisons using the Tukey HSD test were employed due to its ability to effectively control the family-wise error rate while facilitating comprehensive comparisons among group means. In relation to this, participants in Cluster 1 (“most skilled”) showed the highest levels of the sexual items when compared to participants in the other clusters; that is, these participants indicated the highest scores regarding overall sexual well-being, sexual attractiveness, sexual openness and communication, and sexual satisfaction when compared to the other clusters. The observed clusters, differentiated by sexual well-being items, provide crucial insights into the nuanced dynamics of sexual experiences in aging individuals.

While the statistical analysis yielded robust findings, it is essential to acknowledge potential limitations associated with the assumptions underlying cluster analysis, including the assessment of the number of clusters.

## 4. Discussion

This study aimed to identify clusters that were characterized by varying levels of AtA, sexual satisfaction, and SwL, which supported our hypothesis regarding the relationships between these constructs. The three distinct clusters showed specific characteristics: Cluster 1 (“most skilled”) with high AtA, high sexual satisfaction, and high SwL; Cluster 2 (“least adjusted”) with low levels of AtA and SwL, and moderate sexual satisfaction; and Cluster 3 (“aging strivers”), with moderate levels of AtA and low levels of sexual satisfaction and SwL. The three clusters also exhibit distinct sociodemographic, health, lifestyle characteristics, and variations in terms of sexual well-being items.

Furthermore, results indicated that the three clusters showed specific sociodemographic sexual well-being features, which support our hypothesis regarding the variations in sociodemographic, health, and lifestyle factors and sexuality-related items among the participants in each of the three clusters.

Cluster 1 (“most skilled”) reflected a strong alignment of different variables, with high levels of AtA, sexual satisfaction, and SwL. These participants displayed a comprehensive sense of well-being, encompassing purpose, spirituality, health, stability, social support, fulfillment in both individual and partnered sexual experiences, and a high satisfaction with life. Literature indicated that older adults with higher SwL exhibited more positive health outcomes, such as physical health indicators, health behaviors, and psychosocial well-being in subsequent evaluations [42,43]. Moreover, a higher level of sexual well-being has been correlated with greater overall SwL [12,44]. Indeed, perspectives about the importance of maintaining a satisfying sex life in later years have been leading to relations with psychological variables that subsidize sexual satisfaction [45].

This cluster predominantly comprises older adults in a relationship, mostly from Portugal, and predominantly consisting of older women with varying levels of education. These participants self-perceive themselves as spiritual and consider their general health to be good. Moreover, these older adults show diverse experiences of love and engagement in leisure activities. Literature highlights the influence of education, leisure, and spirituality on older adults’ sexual well-being since these factors play a crucial role in the cultural context of older individuals’ sexual perspectives and their sexual satisfaction [46,47,48,49].

Cluster 1 is also distinguished by its higher scores on sexual well-being items. Within this cluster, individuals consistently express a profound sense of positive sexual experiences, marking a noteworthy level of satisfaction with their sexual lives. Their sense of sexual well-being encompasses positive sexual attractiveness, open communication about sex, and overall satisfaction. This cluster represents individuals who find contentment and fulfillment in their sexual experiences, and their positive self-perception contributes to their robust sexual well-being. The literature indicates that open communication about sexuality is strongly associated with higher levels of sexual satisfaction [50]. In a study conducted by Velten and Margraf [51], various factors contributing to sexual satisfaction in stable relationships included partner-related aspects, relationship dynamics, effective sexual communication, and the individual’s SwL.

Participants in Cluster 1 may present higher levels of all variables due to their diverse experiences of love, engagement in leisure activities, and self-perceived spirituality, which contribute to their sense of fulfillment. Additionally, their positive sexual experiences and open communication about sex highlight the importance of interpersonal dynamics and cultural attitudes toward sexuality in shaping sexual satisfaction and overall well-being in older adulthood.

Participants in Cluster 2 (“least adjusted”) demonstrated low AtA, moderate sexual satisfaction, and relatively low SwL. Despite facing challenges, these participants are motivated to find a balance between relatively positive sexual satisfaction and overall satisfaction and adjustment. Studies indicate that SwL played a significant role in predicting AtA [7]. Furthermore, sexual well-being and experiencing happiness and SwL were linked to aging well [44,52].

The second cluster includes both Portuguese and Spanish older adults, with a slightly higher proportion of women with varied educational levels. While this cluster predominantly consists of married participants, it also has a notable percentage of divorced individuals. A significant portion of participants in this cluster identify as spiritual; most report good health and showed varied experiences in love. Interestingly, older adults in this cluster tend not to engage in leisure activities despite the widely recognized benefits of such activities for older people, including the maintenance of cognitive function, physical and mental health, and overall successful aging [47].

Furthermore, older adults in Cluster 2 show a moderate classification of sexual well-being items. Indeed, individuals in this cluster maintain a balanced perspective of their sexual experiences, and their assessment of sexual attractiveness is moderate, reflecting their nuanced perception of their sexual appeal. Considering the average levels of sexual openness and communication, as well as moderate satisfaction with sexual experiences observed in this group, further exploration is needed to understand the underlying factors influencing these aspects of sexual well-being and how they relate to overall SwL. Studies show that successful aging is significantly influenced by sexual desire and being sexually satisfied [23,53,54], and sexual expression may be beneficial to physical and mental health [55]. Additionally, the multifaceted concept of positive attractiveness, which encompasses aspects such as body appreciation and acceptance, has been shown to have an impact on sexual well-being [56,57].

In Cluster 2, despite lower AtA and SwL, individuals maintain moderate levels of sexual satisfaction. Their balanced perspective on sexual experiences suggests a nuanced psychological resilience, emphasizing the importance of individual coping mechanisms in navigating aging-related challenges.

Older participants in Cluster 3 (“aging strivers”) show moderate AtA, low sexual satisfaction, and relatively low SwL. These older adults show a balance between AtA and experiencing limited sexual satisfaction. Their overall SwL is comparatively lower, indicating room for improvement. Prior research revealed a strong connection between sexual satisfaction and contentment in various life domains, such as the ability to take care of oneself, partnership satisfaction, and family life satisfaction [52,58,59]. More recent studies have also demonstrated that sexual satisfaction has been linked to successful aging [44,58], as well as to overall SwL and well-being [60].

Cluster 3 contains a diverse group with an equal mix of Portuguese and Spanish individuals. The majority of participants in this cluster have higher educational attainment and are married, with a significant proportion of widowed individuals. Additionally, these older adults identify themselves as spiritual and report good health. Within this cluster, participants reported positive experiences in love and active engagement in leisure activities. Existing research has indicated a positive relationship between marital status, spirituality, education, and sexual well-being [27,52]. Given the existing evidence indicating a positive association between general well-being, sexual well-being, and SwL, further research on these constructs is warranted to explore how these positive constructs interact and influence each other, especially when considering sociocultural variations across diverse old populations.

Older adults in Cluster 3 exhibited a lower classification of sexual well-being items. Individuals within this cluster express a more restrained sense of satisfaction with their sexual lives. Their sexual attractiveness is relatively low, and their sexual openness and communication are limited, potentially affecting their overall sexual experiences. This cluster included mostly older men with challenges concerning their sexual well-being, likely stemming from issues related to self-perception and communication. In a previous study, among the motivations for a poor sex life among older adults were not having a partner (12.7%), not having desire (9.32%), and physical difficulties (7.62%) [18]. Enhancing sexual well-being may involve efforts to improve self-confidence and foster open conversations about their sexual experiences [27]. Research and clinical data have indicated that couples experiencing sexual difficulties often report poor sexual communication. Positive correlations have been observed between sexual communication, attractiveness, and various facets of sexual function, including desire, arousal, erection, lubrication, orgasm, and reduced pain [61].

In Cluster 3, older adults exhibit moderate AtA but experience low sexual satisfaction and relatively low satisfaction with life. Despite positive attributes such as higher education, marriage, and engagement in leisure activities, they report limited sexual well-being, potentially influenced by issues with self-perception and communication. Efforts to enhance sexual well-being in this group may involve improving self-confidence and fostering open conversations about sexual experiences. Despite similarities in educational attainment and marital status, cultural nuances may also influence perceptions of aging, sexual satisfaction, and overall well-being.

The three clusters were culturally diverse. Cluster 1 comprised mostly Portuguese older adults with high levels of AtA, sexual satisfaction, and SwL. Conversely, Cluster 2 integrated mostly Portuguese participants with moderate sexual satisfaction and lower levels of AtA and SwL. Cluster 3 encompassed mostly Spanish men with moderate levels of AtA and reduced sexual satisfaction and SwL. The significant differences in nationality among the clusters highlight the potential influence of cultural factors on the AtA, SwL, and sexual satisfaction in two different geographic areas in Europe [24,34]. These findings underscore the importance of considering the cultural context in understanding the variability in well-being outcomes among older adults in different geographical regions. The literature highlights the importance of cultural values, such as religious beliefs, in expressing sexuality, attractiveness, intimacy, gender roles, and sexual health [24,34].

Moreover, these results support the multidimensional AtA theoretical framework [32], in which AtA constitutes a multifactor adjustment process with dimensions involving sexuality and intimacy, with implications on well-being, satisfaction with life, and, ultimately, aging well [7,9,32].

This study contains several limitations. While our study comprehensively compiles responses highlighting cultural differences, the oldest-old adults, especially those in their late 80s and 90s were poorly represented. Moreover, there is a lack of diversity in terms of sexual orientation and gender identity within the sample. It is equally critical to distinguish between nationality and cultural identity, which can be nuanced and not necessarily related to a specific nationality. These qualities have specific displays and should be carefully considered, even though there may be situations in which they intersect [24,34]. Connections may also exist between academic education (e.g., risk factors, health promotion) and sexual education, for example, through the promotion of open discussions in the community or psychoeducational programs; however, these two areas cover different topics. Further investigation of these factors in larger-scale and qualitative research may provide insightful information to older populations and health professionals.

Another limitation concerns the reliance on self-reported measures. Older adults could present their perceptions positively, which could further have biased responses, as older individuals might have avoided sharing unfavorable sexual experiences. Because measures were mainly quantitative, these instruments might not fully encompass the intricate experiences of older individuals or possess the necessary precision to detect meaningful changes over time. The use of cross-sectional data limits the ability to establish causality, allowing for unmeasured factors and contextual elements to influence the observed correlations. Factors such as interpersonal relationships, religious beliefs, and access to healthcare could significantly shape perceptions of sexual well-being among older adults. Additionally, cluster analysis primarily serves an exploratory purpose, and different clustering strategies might yield distinct outcomes. It is also important to recognize that the assessment of clusters relies on predefined criteria, which may not fully capture the complexity of the data. Additionally, due to the limited existing research in this field, making potential comparisons based on demographics and outcomes became a challenging endeavor. These limitations underscore the need for cautious interpretation and suggest avenues for future research to address these methodological constraints. Future studies could consider incorporating qualitative data and longitudinal designs to provide deeper insights into the dynamics of aging, sexual satisfaction, and overall well-being among older adults.

Notwithstanding these limitations, our findings have significant implications for both policy development and practical applications in the field of public health. Public health practitioners can prioritize interventions aimed at promoting sexual well-being and overall satisfaction with life among older adults, addressing barriers to sexual communication and openness. In relation to this, psychoeducational interventions may strongly contribute to sharing difficulties and building important resources for older adults and public health practitioners dealing with these. Moreover, policymakers could integrate sexual health education into existing aging programs, while community leaders can facilitate open discussions in the community and promote clear information that endorses healthy attitudes toward sexuality, risk prevention, and, ultimately, aging well. Indeed, prior research has already highlighted that a substantial number of older adults, regardless of gender, often refrain from seeking help for their sexual issues [12]. This study emphasizes the relevance of knowing more about older adults’ sexual well-being and how it relates to SwL and AtA from a cultural perspective. There is a pressing need for healthcare providers to take a proactive role in assisting older adults in achieving positive sexual experiences [24]. These findings not only contribute to academic understanding but also have practical implications. Up to now, interventions rarely focus on sexual well-being and even less on the relation between sexual well-being and aging well [58]. In this context, these results can be an important means for guiding healthcare providers and policymakers in developing tailored interventions to promote a comprehensive approach to well-being among older adults, which includes targeted support programs and community interventions focused on the interaction between sexual satisfaction, adjustment to aging, and overall satisfaction with life.

This study sheds light on the intricate relationship between sexual satisfaction, AtA, and SwL. Our findings indicate that older adults approach these constructs differently. Hence, future interventions with older adults from different cultural contexts should be tailored to their specific needs. Promoting sexual well-being and providing resources to support sexual functioning hold the potential to enhance overall SwL and AtA among older adults. Similarly, initiatives focused on nurturing positive perceptions of AtA and fortifying resilience against age-related changes could facilitate sexual well-being, ultimately contributing to aging well.

In addition to declining health, multiple factors influence sexual activity and satisfaction in later life, as indicated by prior research [24]. Effectively addressing sexual concerns in older adults requires a comprehensive approach that considers not only their needs concerning sexual activity but also various aspects of their lifestyle and the challenges of living well.

This study underscores the critical importance of challenging age-related biases regarding sexuality in older adults. Literature reiterates the importance of sexual well-being for aging well; however, negative stereotypes can hinder the so-needed support available for sexual health concerns and contribute to feelings of isolation and despair. Building knowledge in the sphere of sexual well-being, particularly from a cross-cultural perspective, plays a pivotal role in combating detrimental social stereotypes. These findings shed light on recognizing the strong connection between sexual satisfaction and AtA and SwL has the potential to reshape attitudes, fostering inclusivity that transcends age boundaries. Moreover, this study highlighted the importance of acknowledging the culturally diverse experiences and needs of older adults.

Older adults perceive and prioritize sexual satisfaction, AtA, and SwL differently. Indeed, older populations are demographic heterogeneous, encompassing individuals from different cultural backgrounds, varying socioeconomic statuses, and distinct health conditions. Acknowledging and appreciating this diversity are fundamental steps toward formulating interventions and policies that effectively cater to the unique needs of the various subgroups within the older adult population.

It is imperative to elucidate the intricate relationships among the variables of sexuality, aging, and satisfaction while concurrently investigating intercultural variations [62,63]. The significance of conducting comprehensive cross-cultural studies that scrutinize these dynamics across diverse cultural contexts cannot be overstated [6,7,31,58]. Cultural nuances significantly shape individuals’ perceptions and experiences of aging and sexuality, influencing the generalizability of our findings. By undertaking a study with two different older samples, we aimed to explore cultural differences and to understand how the assessment of these cultural influences is crucial for interpreting and applying our research findings appropriately within their contexts. Considering the cross-cultural nature of our study, it would be advantageous for future research to explore larger older cohorts to identify patterns and assess their stability or evolution over time. Furthermore, considering the anticipated generational differences due to evolving lifestyles, it is important to account for potential shifts in the perceptions of sexual well-being along the life cycle [34,63].

Future approaches to older adults from various cultures benefit from a tailored comprehension of sexual well-being, AtA, and SwL [63]. Different cultures ascribe unique meanings to these concepts and, thus, a nuanced understanding is crucial [33,63].

## 5. Conclusions

In sum, there is a significant shortage of cross-cultural research on these topics, particularly utilizing cluster analysis. This study intended to fill this gap by exploring in detail three patterns of the intricate relation between sexual well-being, AtA, and SwL among older adults. Cluster 1 (“most skilled”) embodied participants with a positive alignment, with high levels of AtA, sexual satisfaction, and SwL. Cluster 2 (“least adjusted”) showed participants with low AtA, moderate sexual satisfaction, and relatively low SwL. Finally, participants from Cluster 3 (“aging strivers”) placed a central focus on AtA, while presenting low sexual satisfaction and SwL. These participants showed significant challenges to their sexual well-being. These findings contributed to scientific knowledge in an insufficient research area, with inconsistencies within the existing literature. Future interventions and policy programs with different cultures and older populations benefit from including sexual well-being, in all its complexity, as an integral and inseparable dimension of the lives of older individuals. By doing so, older adults may be truly empowered to embrace their sexual well-being as an enduring and meaningful part of their aging well. In conclusion, our study adds to the literature by exploring the importance of addressing the multifaceted interaction of sexual satisfaction, adjustment to aging, and overall well-being among older adults, advocating for tailored interventions and further research in multi-cultural contexts, which actively include sexual variables and its relation to satisfaction with life and AtA, among older populations.

## Figures and Tables

**Table 1:** Sociodemographic and health characteristics of the participants.

Characteristics	Portuguese 177 (54.3%)	Spanish 149 (45.7%)	Total (%) 326 (100%)
Age, Average ± SD			M = 70.59 ± 5.24
Gender, *n* (%)			
Female	88 (49.7%)	80 (53.7%)	168 (51.5%)
Male	89 (50.3%)	69 (46.3%)	158 (48.5%)
Educational level, *n* (%)			
<Secondary	118 (66.7%)	5 (3.4%)	123 (37.7%)
Secondary	19 (10.7%)	21 (14.1%)	40 (12.3%)
>Secondary	40 (22.6%)	123 (82.5%)	163 (50.0%)
Marital status, *n* (%)			
Single	15 (8.5%)	8 (5.4%)	23 (7.1%)
Married/de facto union	95 (53.7%)	110 (73.8%)	205 (62.9%)
Divorced/separated	20 (11.3%)	16 (10.7%)	36 (11.0%)
Widow	47 (26.6%)	15 (10.1%)	62 (19.0%)
Household, *n* (%)			
Living with someone	125 (70.6%)	133 (89.3%)	258 (79.1%)
Living alone	52 (23.4%)	16 (10.7%)	68 (20.9%)
Family Annual Income, *n* (%)			
≤25,000 €	119 (67.2%)	41 (27.5%)	160 (49.1%)
>25,000 €	58 (32.8%)	108 (72.5%)	166 (50.9%)
Perceived health, *n* (%)			
Good	132 (74.6%)	133 (89.3%)	265 (81.3%)
Poor	45 (25.4%)	16 (10.7%)	61 (18.7%)

**Table 2:** Clusters centers, frequencies, and *F* statistics for each variable.

	Cluster 1 (*n* = 26)	Cluster 2 (*n* = 115)	Cluster 3 (*n* = 185)	*F*
1. Sense of purpose and ambitions	2.058	0.300	0.475	161.274 ***
2. Zest and spirituality	2.269	0.213	0.451	192.719 ***
3. Body and health	2.642	0.067	0.413	309.887 ***
4. Aging in place and stability	2.585	0.031	0.383	262.061 ***
5. Social support	2.006	0.414	0.539	196.478 ***
6. Ego-centered sexual satisfaction	1.053	0.726	0.599	149.102 ***
7. Partner/sexual activity-centered sexual satisfaction	0.967	0.748	0.601	148.255 ***
8. Satisfaction with life	1.239	0.484	0.475	81.302 ***

*** *p* < 0.001.

**Table 3:** Three cluster solutions according to sociodemographic and lifestyle characteristics.

	*N*	*Cluster 1* *High AtA/High SS/High SwL* *n = 26 (%)*	*Cluster 2* *Low AtA/Moderate SS/Low SwL* *n = 115 (%)*	*Cluster 3* *Moderate AtA/Low SS/Low SwL* *n = 185 (%)*	*χ* ^2^	*df*	*sig.*
Nationality							
Portuguese	177	24 (92.3%)	63 (54.8%)	90 (48.6%)	17.572	2	<0.001
Spanish	149	2 (7.7%)	52 (45.2%)	95 (51.4%)			
Biological sex							
Female	168	13 (50.0%)	67 (58.3%)	88 (47.6%)	3.273	2	0.195
Male	158	13 (50.0%)	48 (41.7%)	97 (52.4%)			
Educational level							
<Secondary	123	23 (88.5%)	47 (40.9%)	53 (28.6%)	45.111	12	<0.001
Secondary	40	0 (0.0%)	13 (11.3%)	27 (14.6%)			
>Secondary	163	3 (11.5%)	55 (47.8%)	105 (56.8%)			
Marital status							
Single	23	4 (15.4%)	8 (7.0%)	11 (5.9%)	45.225	6	<0.001
Married/de facto union	205	17 (65.4%)	69 (60.0%)	119 (64.3%)			
Divorced/separated	36	2 (7.7%)	16 (13.9%)	18 (9.7%)			
Widow	62	3 (11.5%)	22 (19.1%)	37 (20.0%)			
Spiritual person					2.646	2	0.266
Yes	212	19 (73.1%)	69 (60.0%)	124 (67.0%)			
No	114	7 (26.9%)	46 (40.0%)	61 (33.0%)			
Perceived general health					29.556	2	<0.001
Good	265	20 (76.9%)	80 (69.6%)	164 (88.6%)			
Poor	61	6 (23.1%)	35 (30.4%)	21 (11.4%)			
Love experience in life							
Good	234	21 (80.8%)	64 (55.7%)	134 (72.4%)	43.461	2	<0.001
Bad	92	5 (19.2%)	51 (44.3%)	51 (27.5%)			
Leisure activity							
Yes	161	20 (76.9%)	51 (44.3%)	90 (48.6%)	4.332	2	0.115
No	165	6 (23.1%)	64 (55.7%)	95 (51.4%)			

**Table 4:** Three cluster groups according to sexual well-being items: Descriptives and univariate tests.

	Cluster	*M*	*DP*	*F*	*p*
How do you classify your sexual well-being?	1	4.40 a	1.90	16.987	<0.001
	2	2.94 b	1.69		
	3	2.50 c	1.96		
How do you classify your sexual attractiveness?	1	4.62 a	1.51	23.694	<0.001
	2	3.09 b	3.23		
	3	2.19 b	1.81		
How do you classify your sexual openness and communication?	1	5.15 a	1.54	93.160	<0.001
	2	2.84 b	1.68		
	3	2.19 b	1.83		
How do you classify your sexual satisfaction?	1	4.96 a	1.61	93.233	<0.001
	2	2.68 b	1.48		
	3	2.08 c	1.76		

Note: Measures marked with different letters differ statistically, at the level of α < 0.05, according to the Tukey HSD test.

## Data Availability

The data presented in this study are available on request from the corresponding author. The data are not publicly available due to privacy and ethical restrictions.
